# Supplementary figures and images for: Hotspot of Exotic Benthic Marine Invertebrates Discovered in the Tropical East Atlantic: DNA Barcoding Insights From the Bijagós Archipelago, Guinea‐Bissau
Source: Ecol Evol. 2025 Mar 7;15(3):e70964. doi: 10.1002/ece3.70964 (PMC11886419; doi:10.1002/ece3.70964)

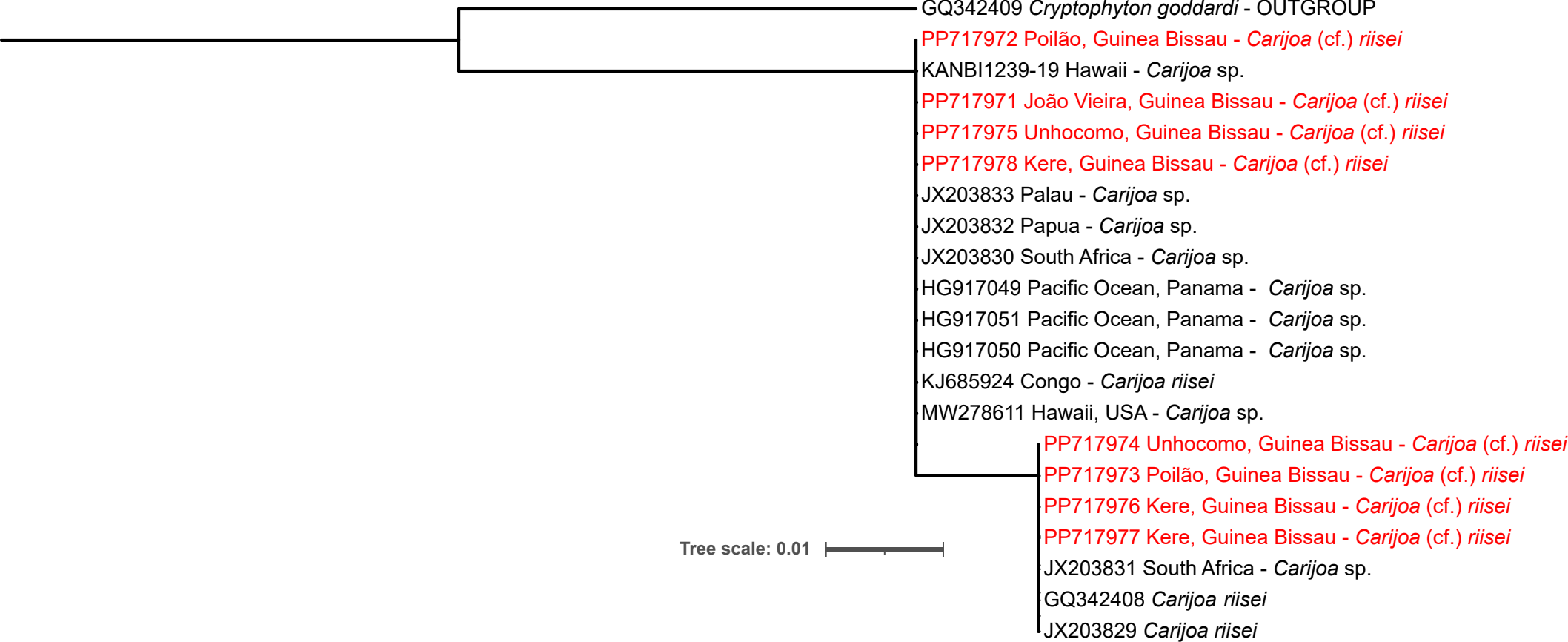

Supplement: Supplementary file 1 — Figure S1 Maximum‐likelihood phylogenetic tree (COI marker) of Carijoa (s.l.) species. [file ECE3-15-e70964-s003.pdf]

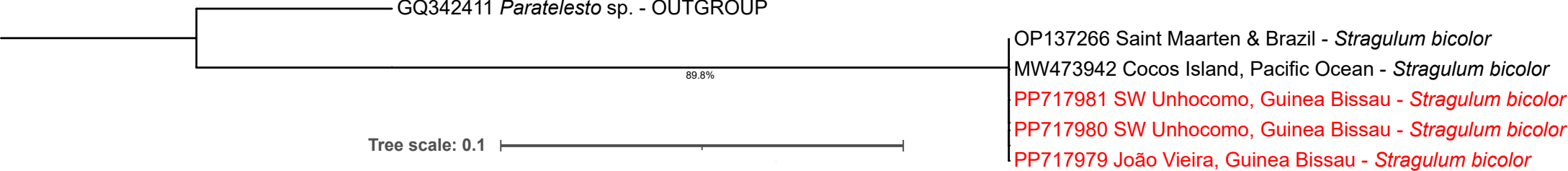

Supplement: Supplementary file 2 — Figure S2 Maximum‐likelihood phylogenetic tree (COI marker) of Stragulum bicolor. [file ECE3-15-e70964-s002.pdf]

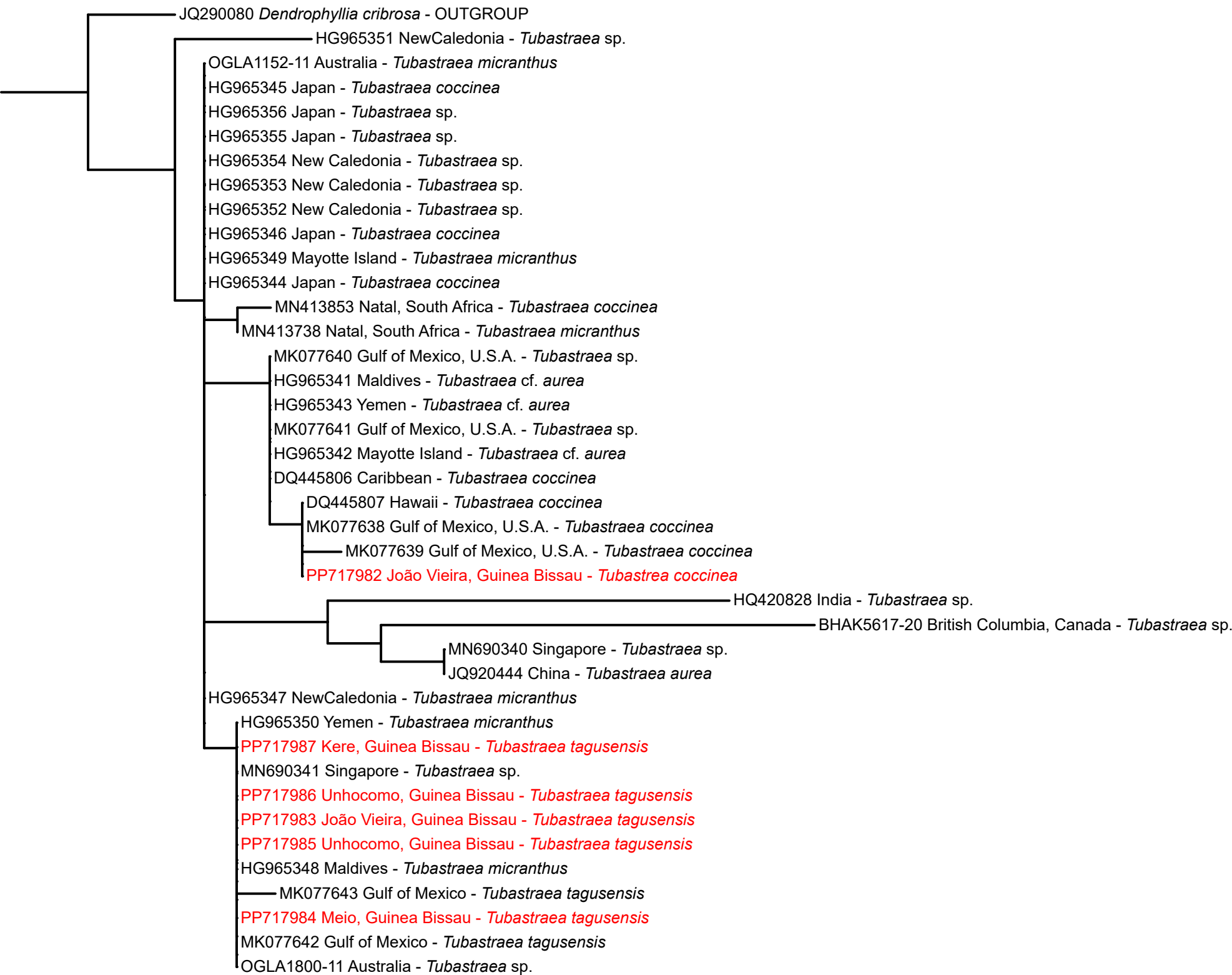

Supplement: Supplementary file 3 — Figure S3 Maximum‐likelihood phylogenetic tree (COI marker) of Tubastraea species. [file ECE3-15-e70964-s017.pdf]

MH029856 *Turritopsis* sp. - OUTGROUP

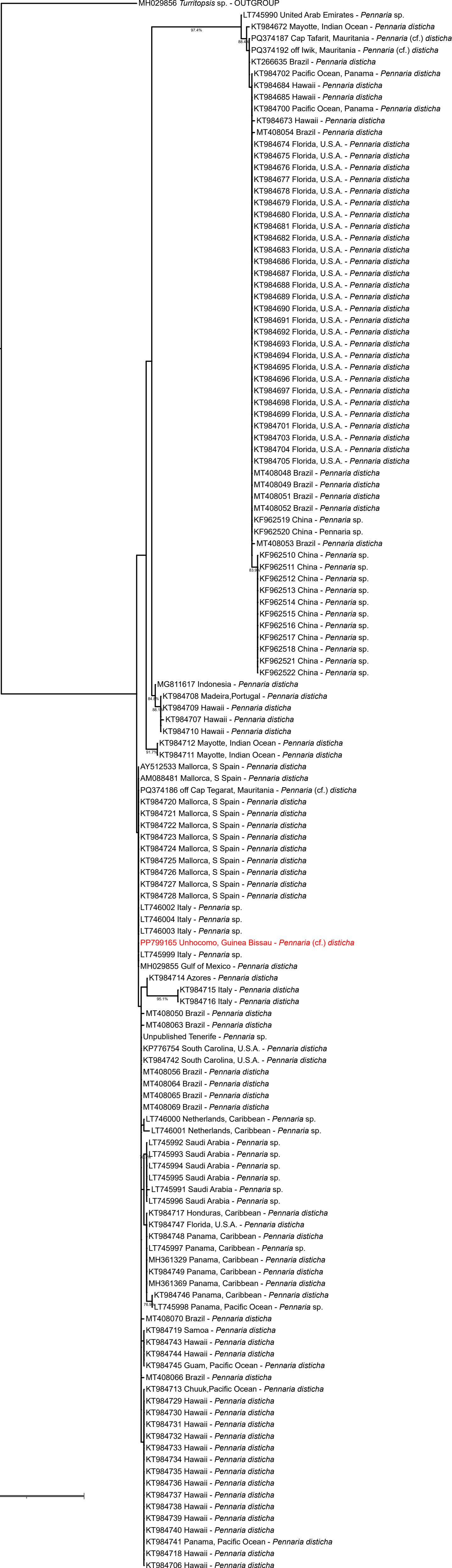

Tree scale: 0.1

Supplement: Supplementary file 4 — Figure S4 Maximum‐likelihood phylogenetic tree (16S marker) of Pennaria species. [file ECE3-15-e70964-s011.pdf]

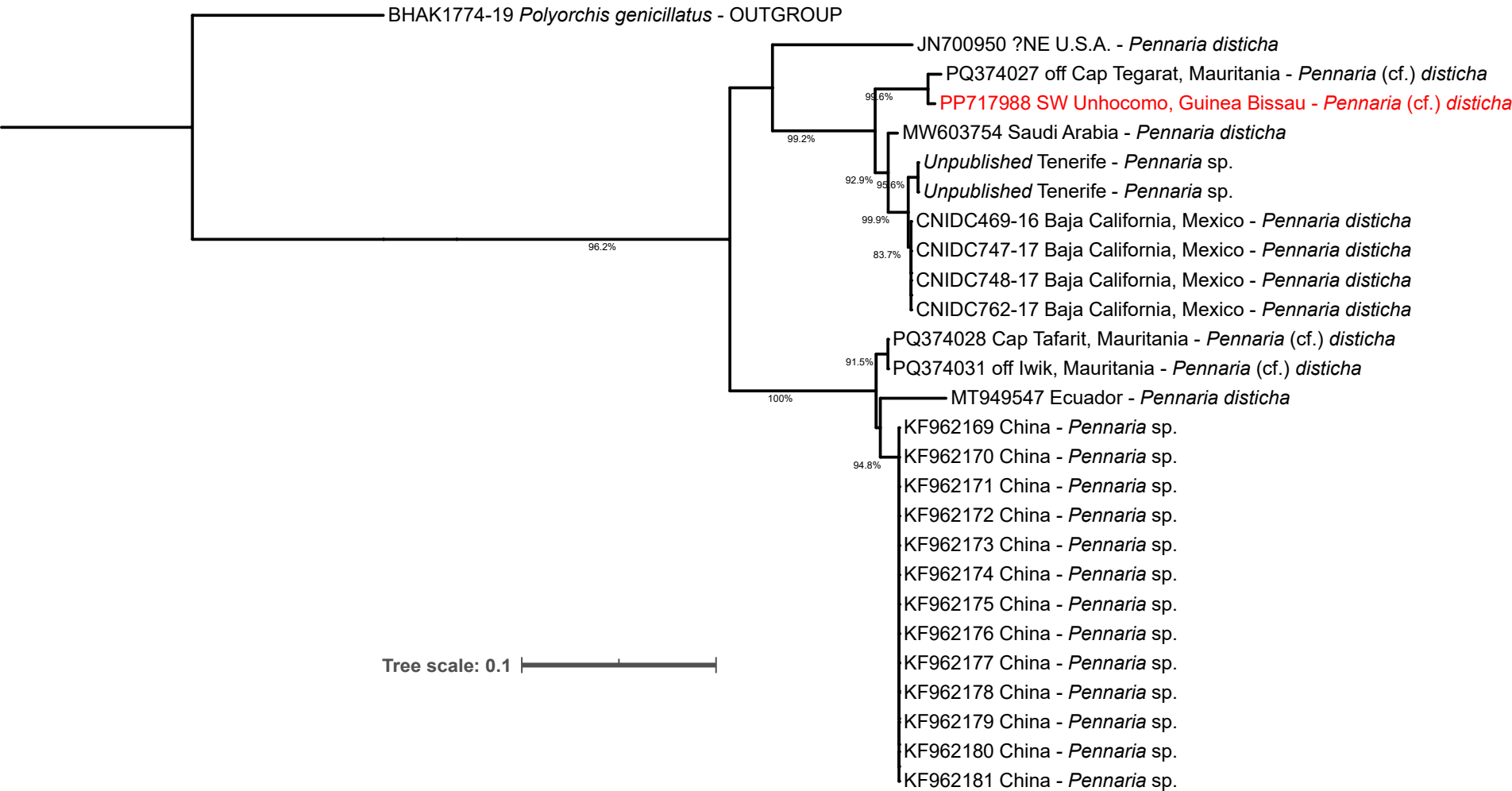

Supplement: Supplementary file 5 — Figure S5 Maximum‐likelihood phylogenetic tree (COI marker) of Pennaria species. [file ECE3-15-e70964-s018.pdf]

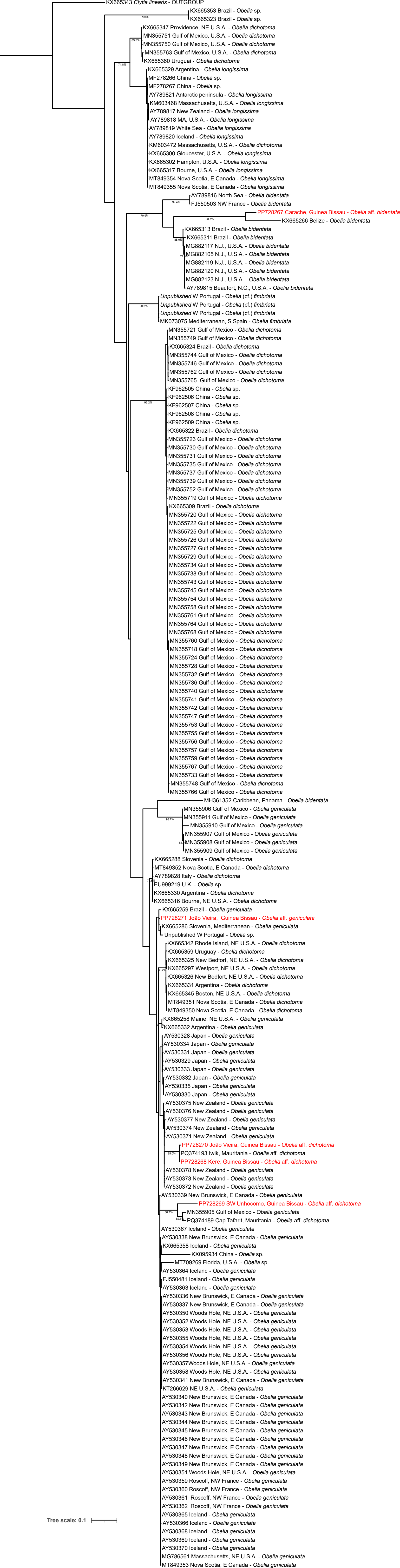

Supplement: Supplementary file 7 — Figure S7 Maximum‐likelihood phylogenetic tree (16S marker) of Obelia species. [file ECE3-15-e70964-s009.pdf]

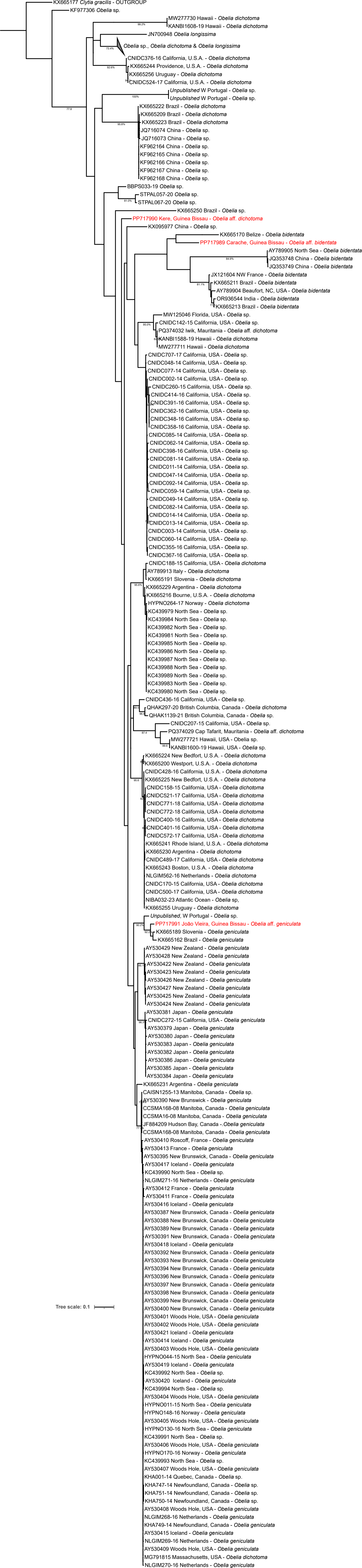

Supplement: Supplementary file 8 — Figure S8 Maximum‐likelihood phylogenetic tree (COI marker) of Obelia species. [file ECE3-15-e70964-s012.pdf]

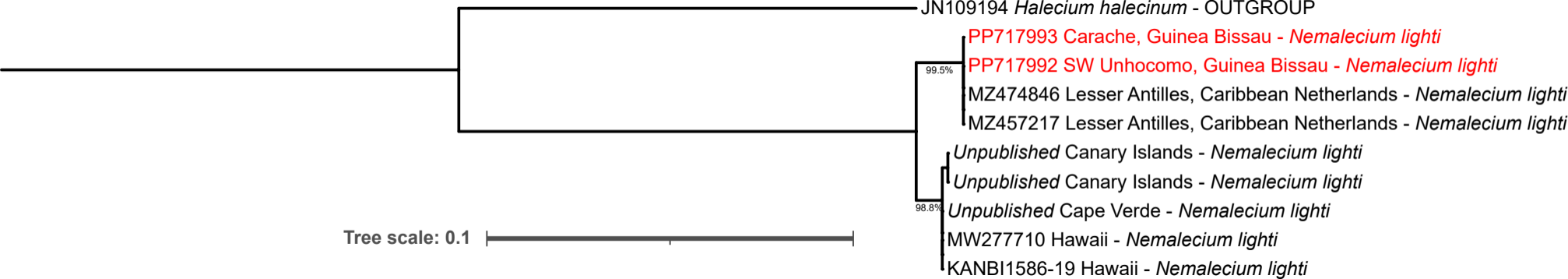

Supplement: Supplementary file 9 — Figure S9 Maximum‐likelihood phylogenetic tree (COI marker) of Nemalecium lighti. [file ECE3-15-e70964-s004.pdf]

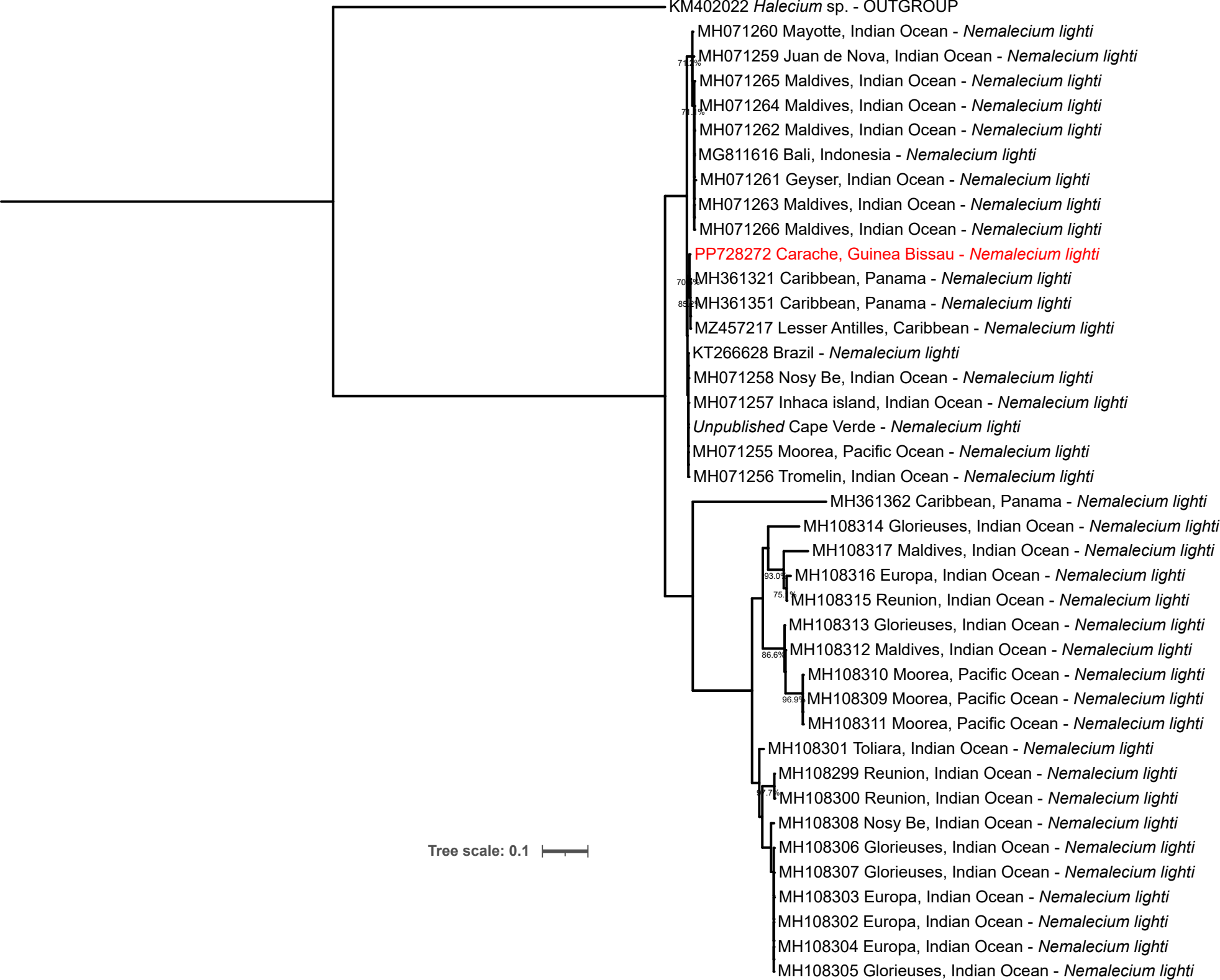

Supplement: Supplementary file 10 — Figure S10 Maximum‐likelihood phylogenetic tree (16S marker) of Nemalecium lighti. [file ECE3-15-e70964-s021.pdf]

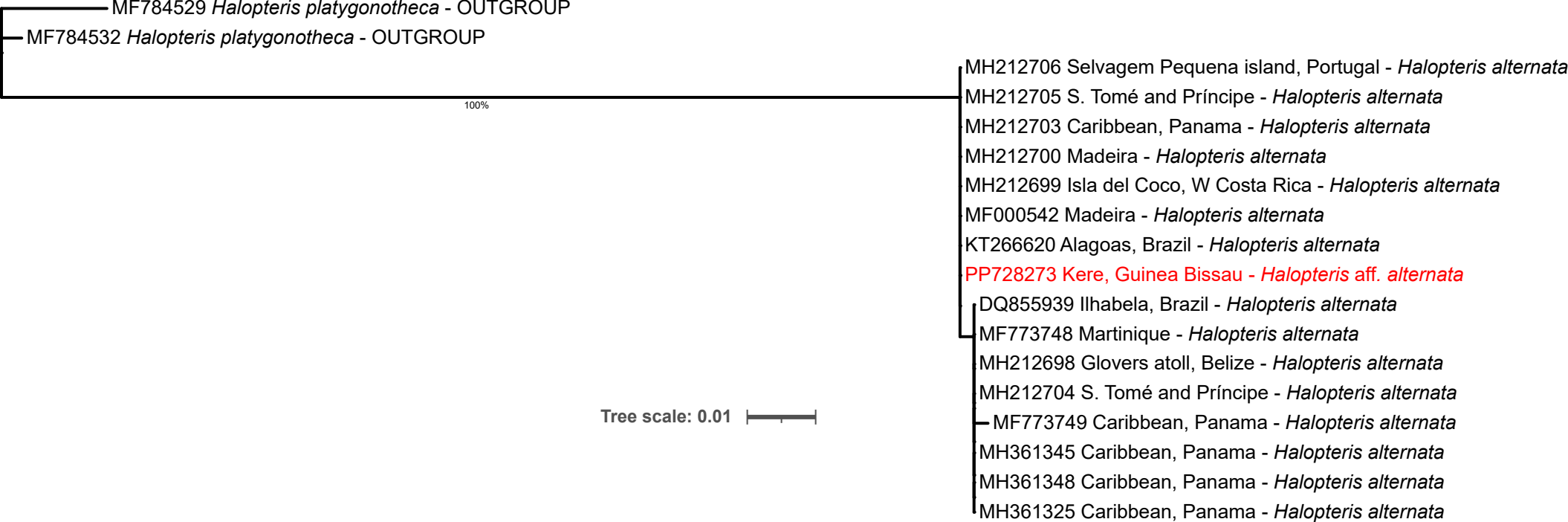

Supplement: Supplementary file 11 — Figure S11 Maximum‐likelihood phylogenetic tree (16S marker) of Halopteris aff. alternata. [file ECE3-15-e70964-s010.pdf]

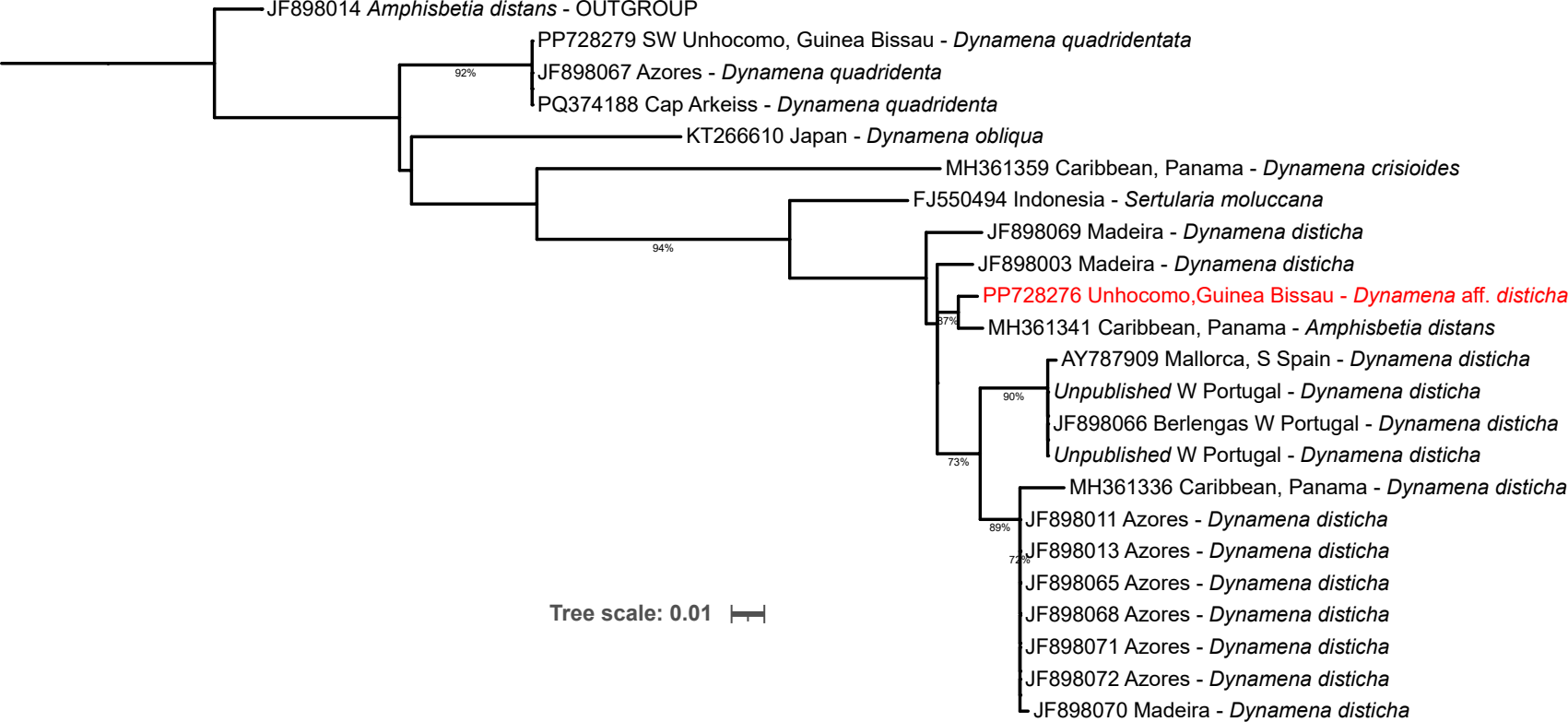

Supplement: Supplementary file 13 — Figure S13 Maximum‐likelihood phylogenetic tree (16S marker) of Dynamena species. [file ECE3-15-e70964-s016.pdf]

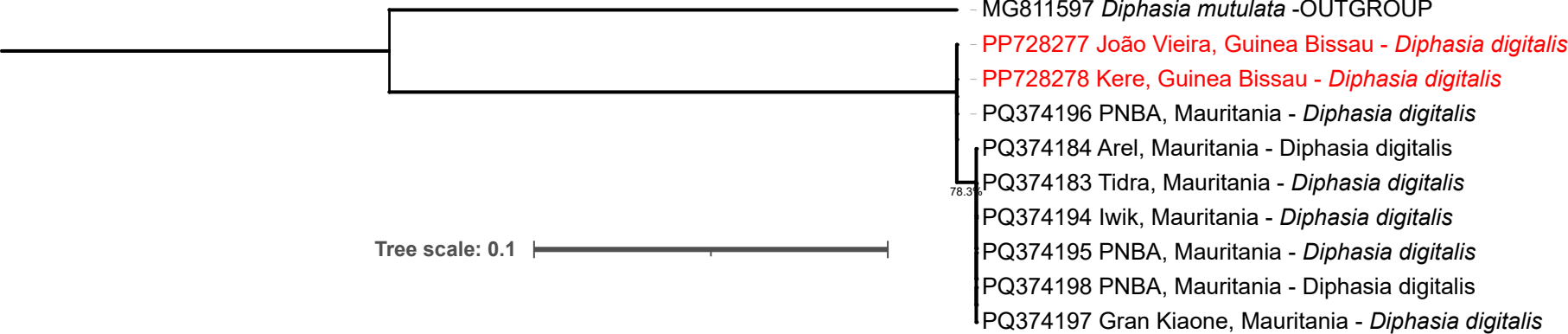

Supplement: Supplementary file 14 — Figure S14 Maximum‐likelihood phylogenetic tree (16S marker) of Dyphasia digitalis. [file ECE3-15-e70964-s014.pdf]

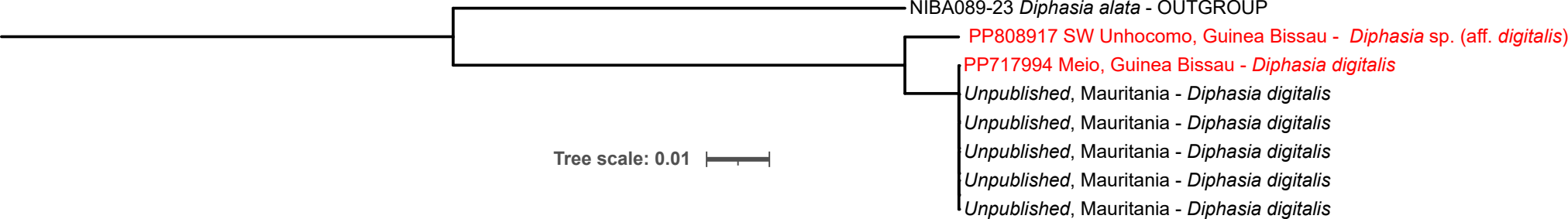

Supplement: Supplementary file 15 — Figure S15 Maximum‐likelihood phylogenetic tree (COI marker) of Dyphasia species. [file ECE3-15-e70964-s023.pdf]

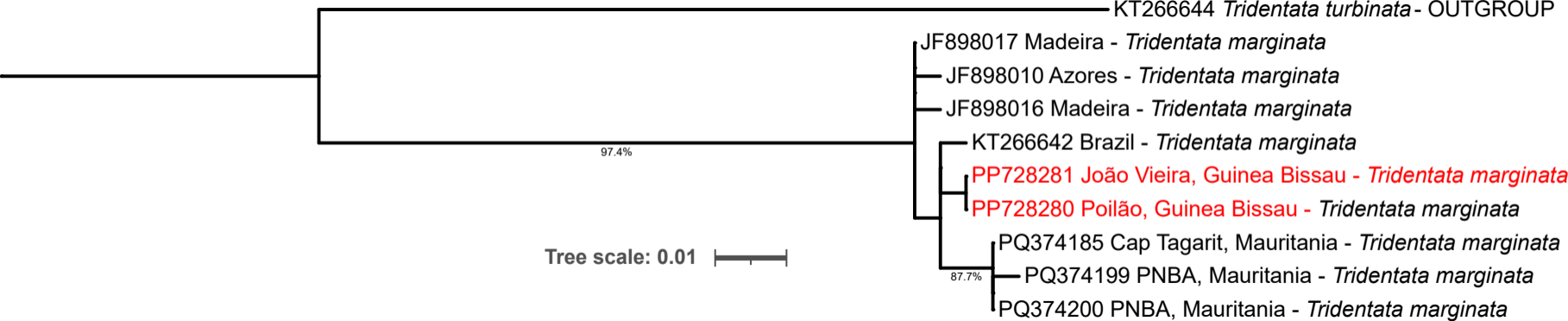

Supplement: Supplementary file 16 — Figure S16 Maximum‐likelihood phylogenetic tree (16Smarker) of Tridentata marginata . [file ECE3-15-e70964-s008.pdf]

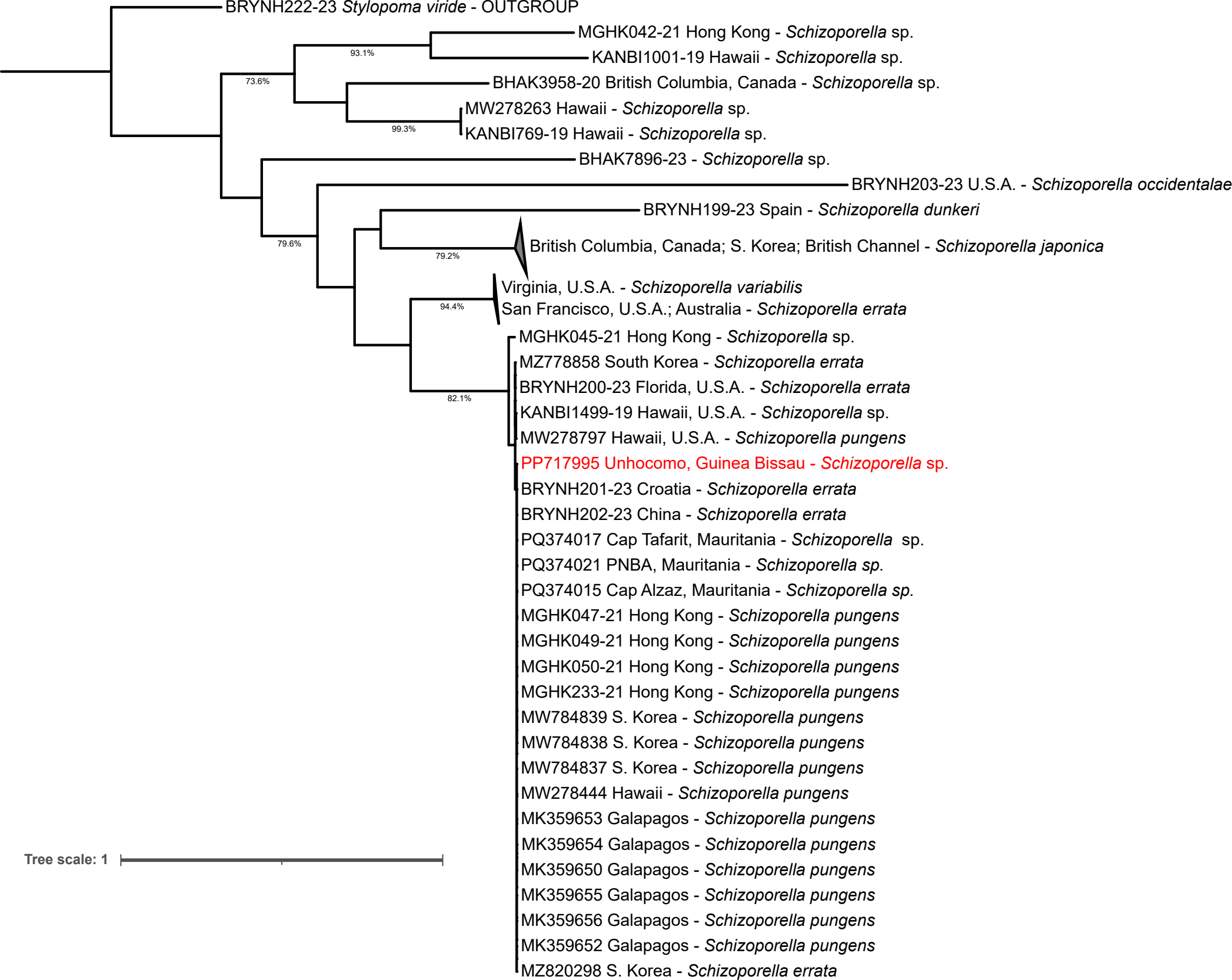

Supplement: Supplementary file 17 — Figure S17 Maximum‐likelihood phylogenetic tree (COI marker) of Schizoporella species. [file ECE3-15-e70964-s020.pdf]

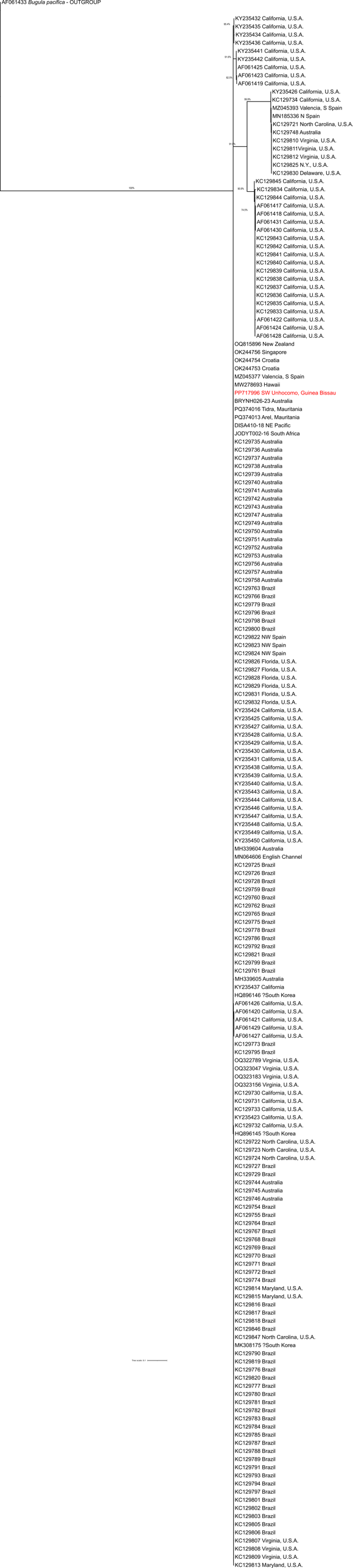

Supplement: Supplementary file 18 — Figure S18 Maximum‐likelihood phylogenetic tree (COI marker) of Bugula neritina . [file ECE3-15-e70964-s001.pdf]

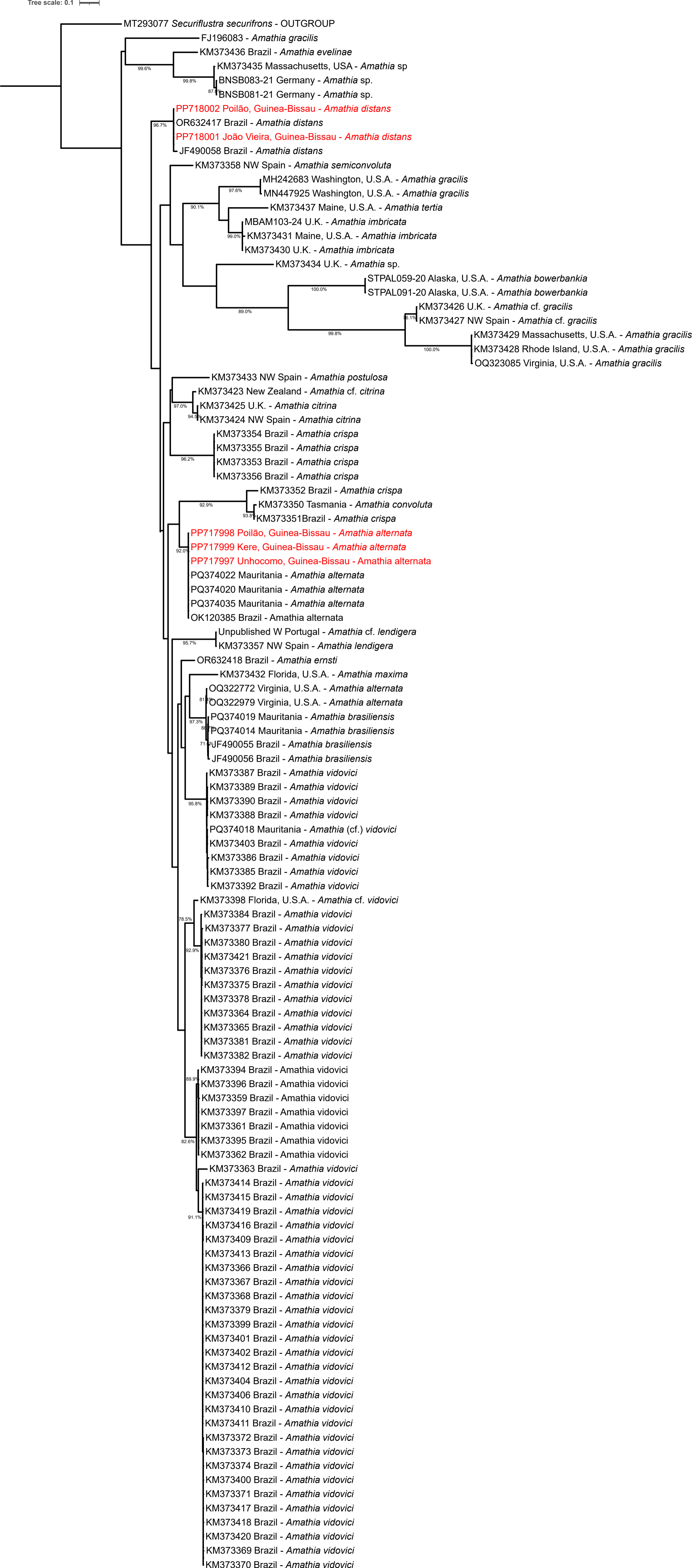

Supplement: Supplementary file 19 — Figure S19 Maximum‐likelihood phylogenetic tree (COI marker) of Amathia species. [file ECE3-15-e70964-s013.pdf]

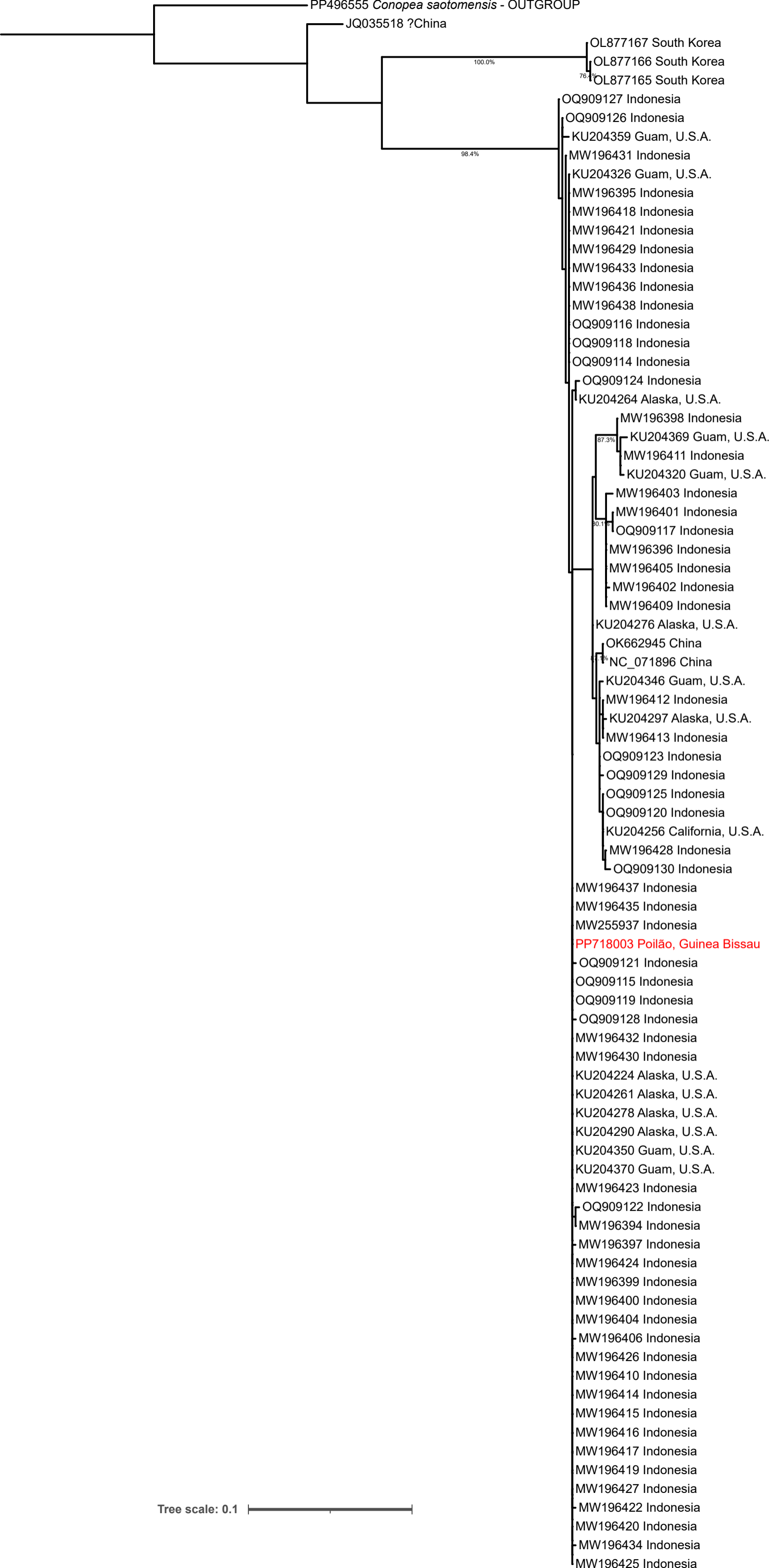

Supplement: Supplementary file 20 — Figure S20 Maximum‐likelihood phylogenetic tree (COI marker) of Amphibalanus reticulatus. [file ECE3-15-e70964-s007.pdf]

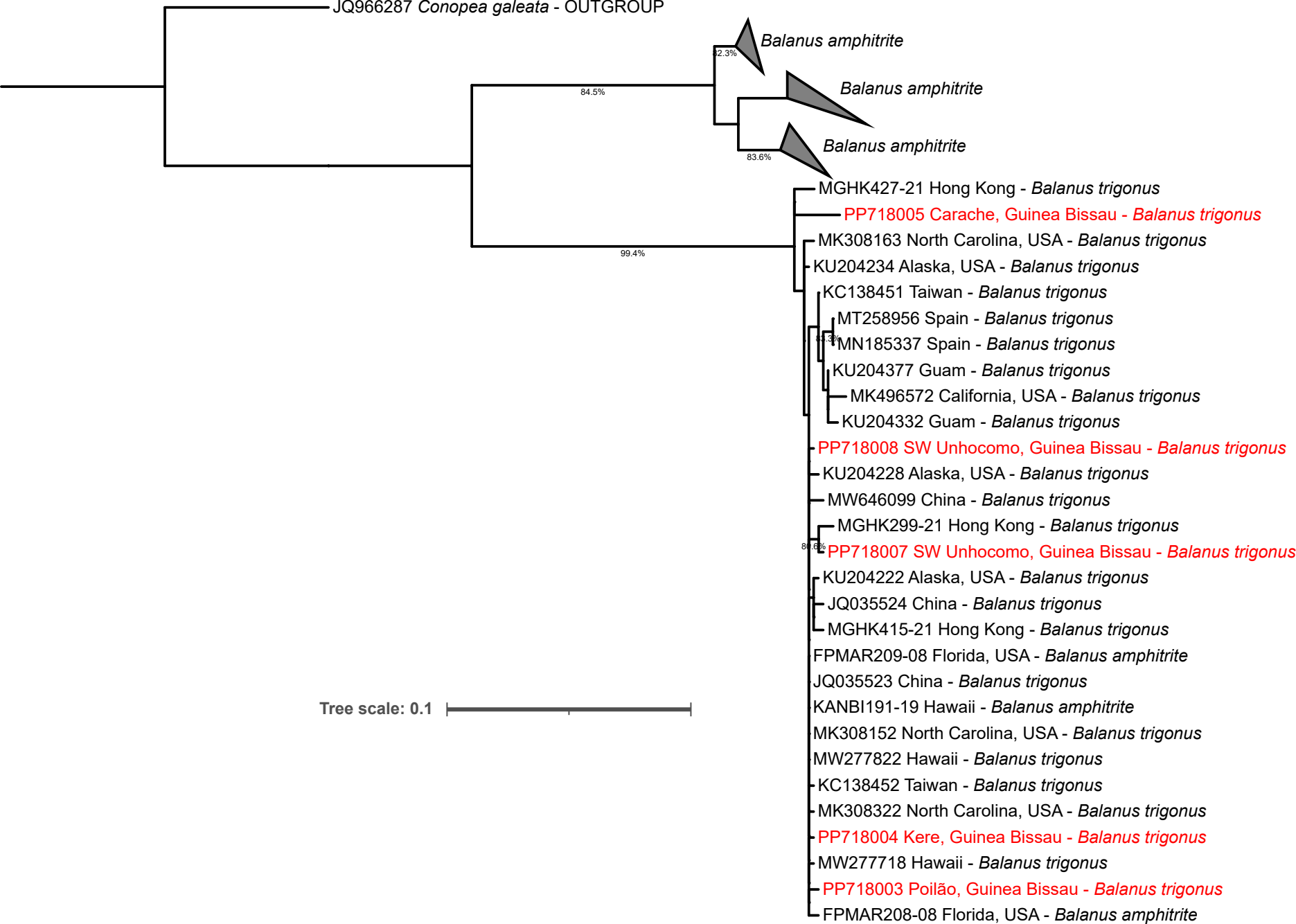

Supplement: Supplementary file 21 — Figure S21 Maximum‐likelihood phylogenetic tree (COI marker) of Balanus species. [file ECE3-15-e70964-s019.pdf]

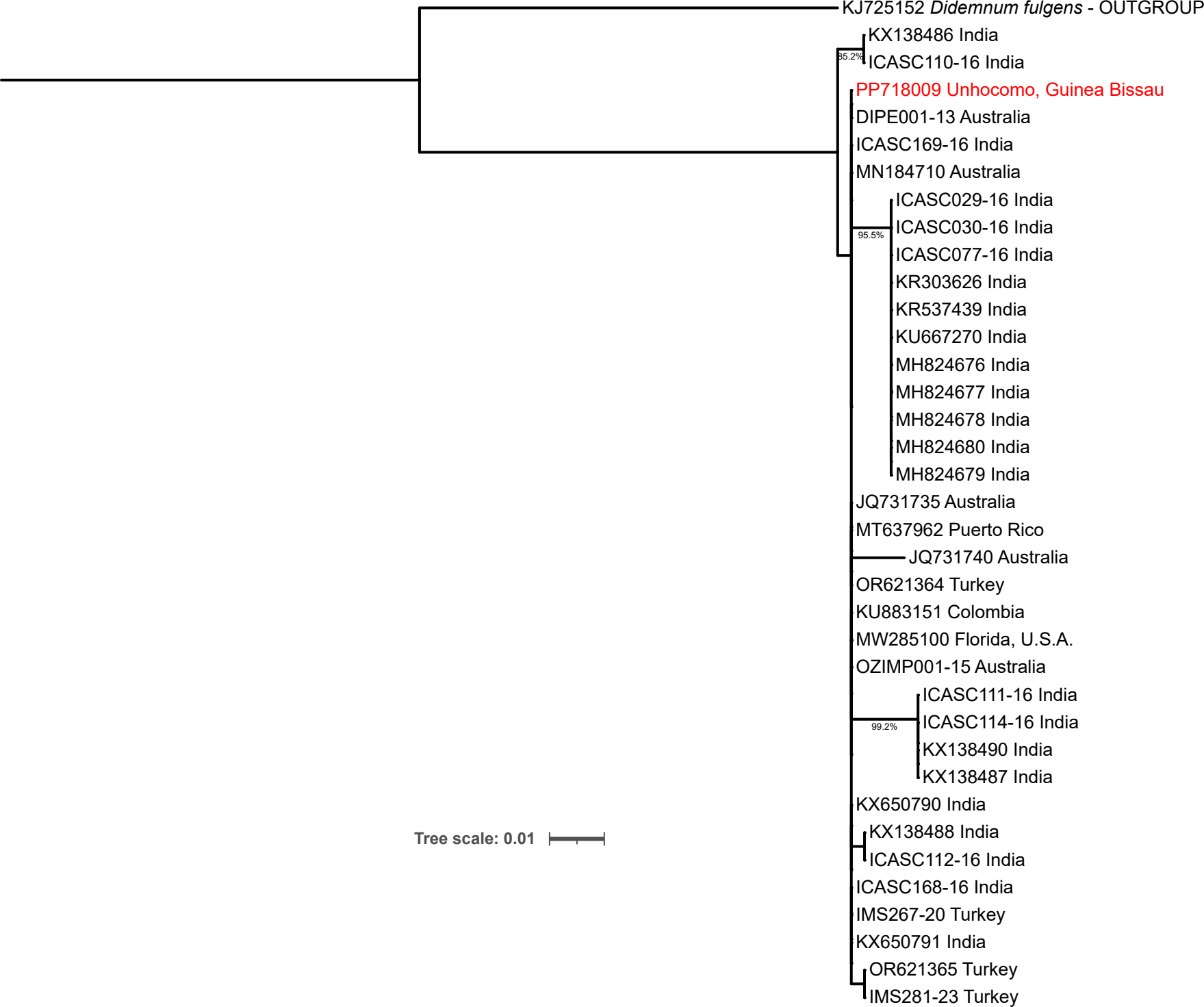

Supplement: Supplementary file 22 — Figure S22 Maximum‐likelihood phylogenetic tree (COI marker) of Didemnum perlucidum. [file ECE3-15-e70964-s015.pdf]

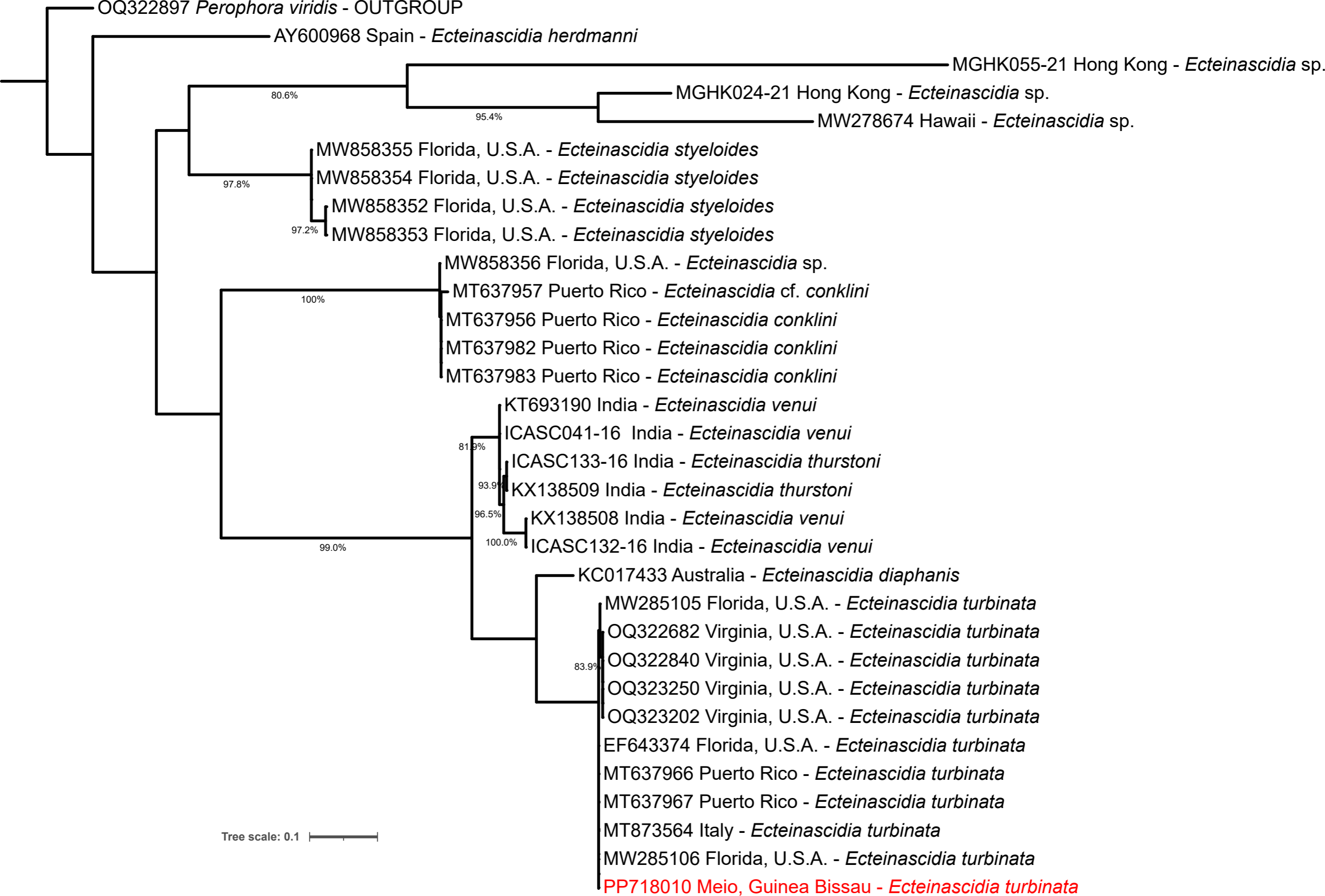

Supplement: Supplementary file 23 — Figure S23 Maximum‐likelihood phylogenetic tree (COI marker) of Ecteinascidia species. [file ECE3-15-e70964-s024.pdf]

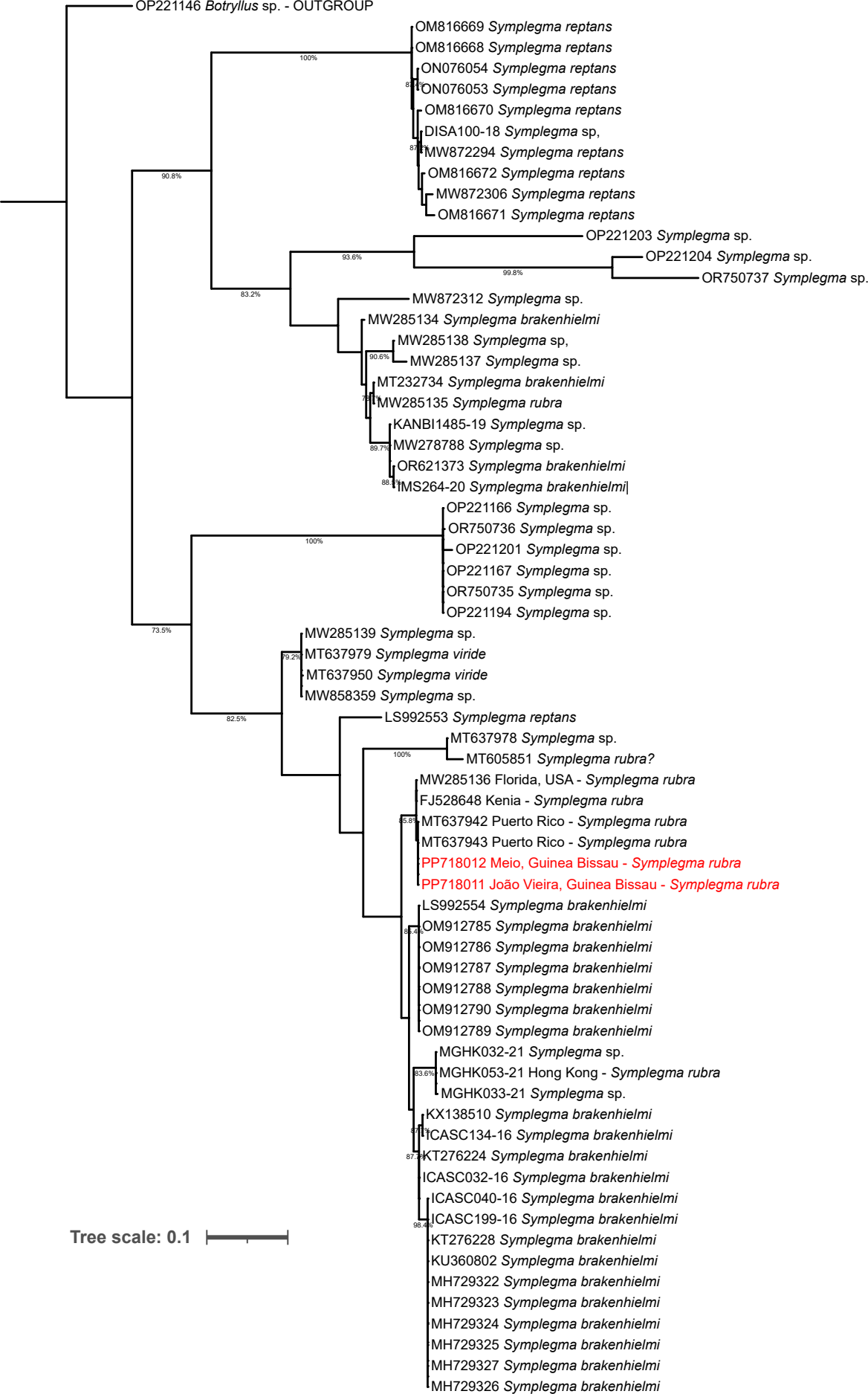

Supplement: Supplementary file 24 — Figure S24 Maximum‐likelihood phylogenetic tree (COI marker) of Symplegma species. [file ECE3-15-e70964-s006.pdf]
