# Supplementary material for: Hotspot of Exotic Benthic Marine Invertebrates Discovered in the Tropical East Atlantic: DNA Barcoding Insights From the Bijagós Archipelago, Guinea‐Bissau
Source: Ecol Evol. 2025 Mar 7;15(3):e70964. doi: 10.1002/ece3.70964 (PMC11886419; doi:10.1002/ece3.70964)
Supplement: Supplementary file 6 — Figure S6 Maximum‐likelihood phylogenetic tree (COI marker) of Macrorhynchia species. [file ECE3-15-e70964-s022.pdf]

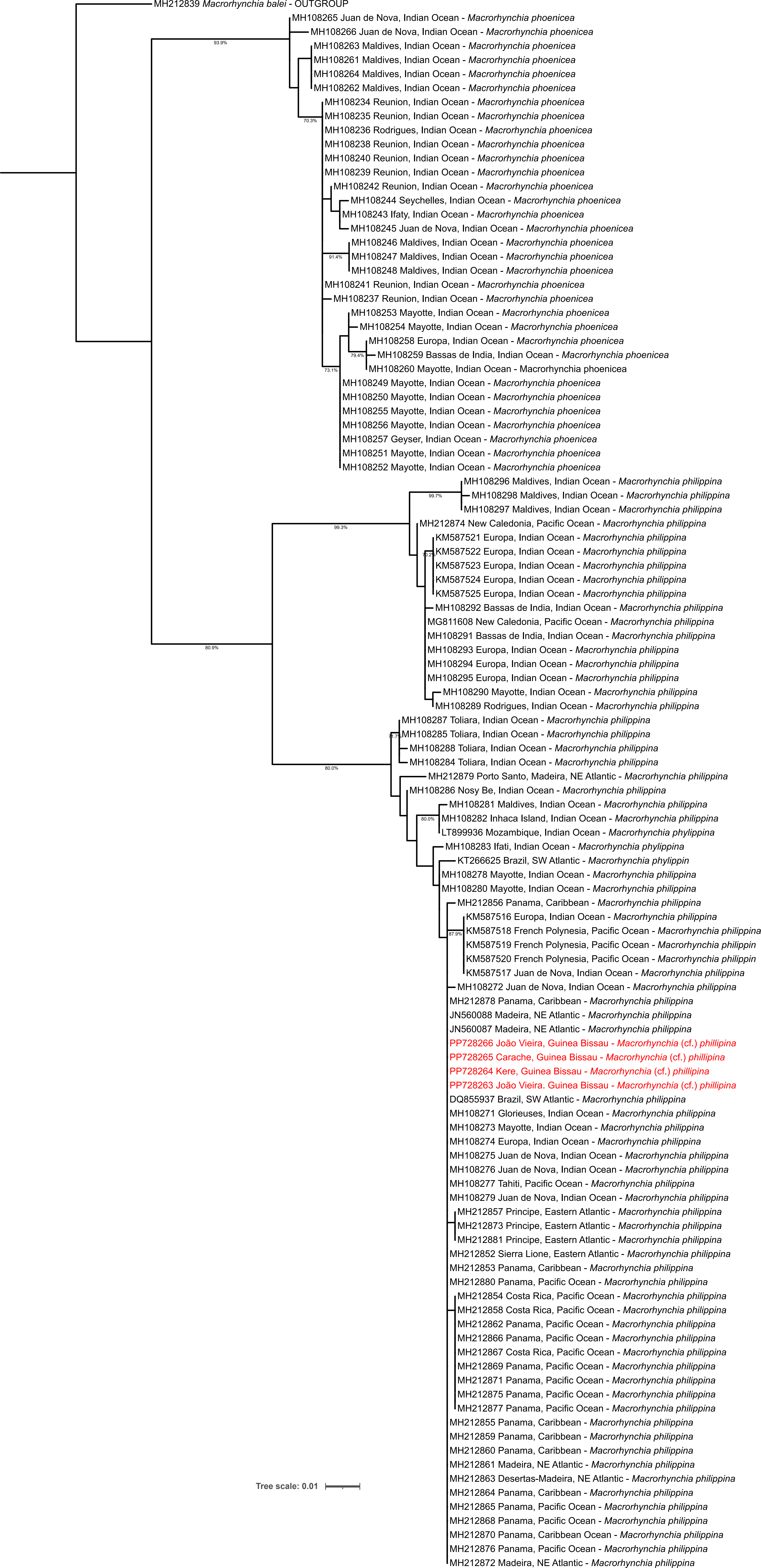

MH212839 *Macrorhynchia balei* - OUTGROUP
MH108265 Juan de Nova, Indian Ocean - *Macrorhynchia phoenicea*
MH108266 Juan de Nova, Indian Ocean - *Macrorhynchia phoenicea*
93.9%
MH108263 Maldives, Indian Ocean - *Macrorhynchia phoenicea*
MH108261 Maldives, Indian Ocean - *Macrorhynchia phoenicea*
MH108264 Maldives, Indian Ocean - *Macrorhynchia phoenicea*
MH108262 Maldives, Indian Ocean - *Macrorhynchia phoenicea*
MH108234 Reunion, Indian Ocean - *Macrorhynchia phoenicea*
MH108235 Reunion, Indian Ocean - *Macrorhynchia phoenicea*
70.3%
MH108236 Rodrigues, Indian Ocean - *Macrorhynchia phoenicea*
MH108238 Reunion, Indian Ocean - *Macrorhynchia phoenicea*
MH108240 Reunion, Indian Ocean - *Macrorhynchia phoenicea*
MH108239 Reunion, Indian Ocean - *Macrorhynchia phoenicea*
MH108242 Reunion, Indian Ocean - *Macrorhynchia phoenicea*
MH108244 Seychelles, Indian Ocean - *Macrorhynchia phoenicea*
MH108243 Ifaty, Indian Ocean - *Macrorhynchia phoenicea*
MH108245 Juan de Nova, Indian Ocean - *Macrorhynchia phoenicea*
MH108246 Maldives, Indian Ocean - *Macrorhynchia phoenicea*
91.4%
MH108247 Maldives, Indian Ocean - *Macrorhynchia phoenicea*
MH108248 Maldives, Indian Ocean - *Macrorhynchia phoenicea*
MH108241 Reunion, Indian Ocean - *Macrorhynchia phoenicea*
MH108237 Reunion, Indian Ocean - *Macrorhynchia phoenicea*
MH108253 Mayotte, Indian Ocean - *Macrorhynchia phoenicea*
MH108254 Mayotte, Indian Ocean - *Macrorhynchia phoenicea*
MH108258 Europa, Indian Ocean - *Macrorhynchia phoenicea*
79.4%
MH108259 Bassas de India, Indian Ocean - *Macrorhynchia phoenicea*
73.1%
MH108260 Mayotte, Indian Ocean - Macrorhynchia phoenicea
MH108249 Mayotte, Indian Ocean - *Macrorhynchia phoenicea*
MH108250 Mayotte, Indian Ocean - *Macrorhynchia phoenicea*
MH108255 Mayotte, Indian Ocean - *Macrorhynchia phoenicea*
MH108256 Mayotte, Indian Ocean - *Macrorhynchia phoenicea*
MH108257 Geyser, Indian Ocean - *Macrorhynchia phoenicea*
MH108251 Mayotte, Indian Ocean - *Macrorhynchia phoenicea*
MH108252 Mayotte, Indian Ocean - *Macrorhynchia phoenicea*
MH108296 Maldives, Indian Ocean - *Macrorhynchia philippina*
99.7%
MH108298 Maldives, Indian Ocean - *Macrorhynchia philippina*
MH108297 Maldives, Indian Ocean - *Macrorhynchia philippina*
99.3%
MH212874 New Caledonia, Pacific Ocean - *Macrorhynchia philippina*
KM587521 Europa, Indian Ocean - *Macrorhynchia philippina*
KM587522 Europa, Indian Ocean - *Macrorhynchia philippina*
KM587523 Europa, Indian Ocean - *Macrorhynchia philippina*
KM587524 Europa, Indian Ocean - *Macrorhynchia philippina*
KM587525 Europa, Indian Ocean - *Macrorhynchia philippina*
MH108292 Bassas de India, Indian Ocean - *Macrorhynchia philippina*
MG811608 New Caledonia, Pacific Ocean - *Macrorhynchia philippina*
80.9%
MH108291 Bassas de India, Indian Ocean - *Macrorhynchia philippina*
MH108293 Europa, Indian Ocean - *Macrorhynchia philippina*
MH108294 Europa, Indian Ocean - *Macrorhynchia philippina*
MH108295 Europa, Indian Ocean - *Macrorhynchia philippina*
MH108290 Mayotte, Indian Ocean - *Macrorhynchia philippina*
MH108289 Rodrigues, Indian Ocean - *Macrorhynchia philippina*
MH108287 Toliara, Indian Ocean - *Macrorhynchia philippina*
MH108285 Toliara, Indian Ocean - *Macrorhynchia philippina*
MH108288 Toliara, Indian Ocean - *Macrorhynchia philippina*
MH108284 Toliara, Indian Ocean - *Macrorhynchia philippina*
80.0%
MH212879 Porto Santo, Madeira, NE Atlantic - *Macrorhynchia philippina*
MH108286 Nosy Be, Indian Ocean - *Macrorhynchia philippina*
MH108281 Maldives, Indian Ocean - *Macrorhynchia philippina*
80.0%
MH108282 Inhaca Island, Indian Ocean - *Macrorhynchia philippina*
LT899936 Mozambique, Indian Ocean - *Macrorhynchia phylippina*
MH108283 Ifati, Indian Ocean - *Macrorhynchia phylippina*
KT266625 Brazil, SW Atlantic - *Macrorhynchia phylippin*
MH108278 Mayotte, Indian Ocean - *Macrorhynchia philippina*
MH108280 Mayotte, Indian Ocean - *Macrorhynchia philippina*
MH212856 Panama, Caribbean - *Macrorhynchia philippina*
KM587516 Europa, Indian Ocean - *Macrorhynchia philippina*
87.9%
KM587518 French Polynesia, Pacific Ocean - *Macrorhynchia philippina*
KM587519 French Polynesia, Pacific Ocean - *Macrorhynchia philippin*
KM587520 French Polynesia, Pacific Ocean - *Macrorhynchia philippin*
KM587517 Juan de Nova, Indian Ocean - *Macrorhynchia philippina*
MH108272 Juan de Nova, Indian Ocean - *Macrorhynchia philippina*
MH212878 Panama, Caribbean - *Macrorhynchia philippina*
JN560088 Madeira, NE Atlantic - *Macrorhynchia philippina*
JN560087 Madeira, NE Atlantic - *Macrorhynchia philippina*
PP728266 João Vieira, Guinea Bissau - *Macrorhynchia* (cf.) *phillipina*
PP728265 Carache, Guinea Bissau - *Macrorhynchia* (cf.) *phillipina*
PP728264 Kere, Guinea Bissau - *Macrorhynchia* (cf.) *phillipina*
PP728263 João Vieira. Guinea Bissau - *Macrorhynchia* (cf.) *phillipina*
DQ855937 Brazil, SW Atlantic - *Macrorhynchia philippina*
MH108271 Glorieuses, Indian Ocean - *Macrorhynchia philippina*
MH108273 Mayotte, Indian Ocean - *Macrorhynchia philippina*
MH108274 Europa, Indian Ocean - *Macrorhynchia philippina*
MH108275 Juan de Nova, Indian Ocean - *Macrorhynchia philippina*
MH108276 Juan de Nova, Indian Ocean - *Macrorhynchia philippina*
MH108277 Tahiti, Pacific Ocean - *Macrorhynchia philippina*
MH108279 Juan de Nova, Indian Ocean - *Macrorhynchia philippina*
MH212857 Principe, Eastern Atlantic - *Macrorhynchia philippina*
MH212873 Principe, Eastern Atlantic - *Macrorhynchia philippina*
MH212881 Principe, Eastern Atlantic - *Macrorhynchia philippina*
MH212852 Sierra Lione, Eastern Atlantic - *Macrorhynchia philippina*
MH212853 Panama, Caribbean - *Macrorhynchia philippina*
MH212880 Panama, Pacific Ocean - *Macrorhynchia philippina*
MH212854 Costa Rica, Pacific Ocean - *Macrorhynchia philippina*
MH212858 Costa Rica, Pacific Ocean - *Macrorhynchia philippina*
MH212862 Panama, Pacific Ocean - *Macrorhynchia philippina*
MH212866 Panama, Pacific Ocean - *Macrorhynchia philippina*
MH212867 Costa Rica, Pacific Ocean - *Macrorhynchia philippina*
MH212869 Panama, Pacific Ocean - *Macrorhynchia philippina*
MH212871 Panama, Pacific Ocean - *Macrorhynchia philippina*
MH212875 Panama, Pacific Ocean - *Macrorhynchia philippina*
MH212877 Panama, Pacific Ocean - *Macrorhynchia philippina*
MH212855 Panama, Caribbean - *Macrorhynchia philippina*
MH212859 Panama, Caribbean - *Macrorhynchia philippina*
MH212860 Panama, Caribbean - *Macrorhynchia philippina*
MH212861 Madeira, NE Atlantic - *Macrorhynchia philippina*
MH212863 Desertas-Madeira, NE Atlantic - *Macrorhynchia philippina*
Tree scale: 0.01
MH212864 Panama, Caribbean - *Macrorhynchia philippina*
MH212865 Panama, Pacific Ocean - *Macrorhynchia philippina*
MH212868 Panama, Pacific Ocean - *Macrorhynchia philippina*
MH212870 Panama, Caribbean Ocean - *Macrorhynchia philippina*
MH212876 Panama, Pacific Ocean - *Macrorhynchia philippina*
MH212872 Madeira, NE Atlantic - *Macrorhynchia philippina*
