# Supplementary material for: Hotspot of Exotic Benthic Marine Invertebrates Discovered in the Tropical East Atlantic: DNA Barcoding Insights From the Bijagós Archipelago, Guinea‐Bissau
Source: Ecol Evol. 2025 Mar 7;15(3):e70964. doi: 10.1002/ece3.70964 (PMC11886419; doi:10.1002/ece3.70964)
Supplement: Supplementary file 12 — Figure S12 Maximum‐likelihood phylogenetic tree (16S marker) of Plumularia species. [file ECE3-15-e70964-s005.pdf]

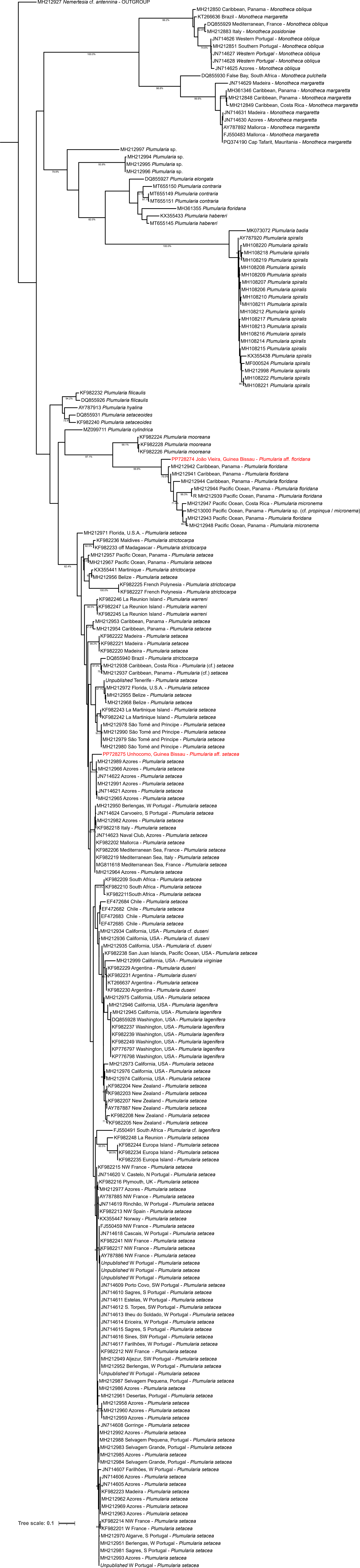

MH212927 Nemertesia cf. antennina - OUTGROUP
MH212850 Caribbean, Panama - Monotheca obliqua
KT266636 Brazil - Monotheca margaretta
DQ855929 Mediterranean, France - Monotheca obliqua
MH212883 Italy - Monotheca posidoniae
JN714626 Western Portugal - Monotheca obliqua
MH212851 Southern Portugal - Monotheca obliqua
JN714627 Western Portugal - Monotheca obliqua
JN714628 Western Portugal - Monotheca obliqua
JN714625 Azores - Monotheca obliqua
DQ855930 False Bay, South Africa - Monotheca pulchella
JN714629 Madeira - Monotheca margaretta
MH361346 Caribbean, Panama - Monotheca margaretta
MH212848 Caribbean, Panama - Monotheca margaretta
MH212849 Caribbean, Costa Rica - Monotheca margaretta
JN714631 Madeira - Monotheca margaretta
JN714630 Azores - Monotheca margaretta
AY787892 Mallorca - Monotheca margaretta
FJ550483 Mallorca - Monotheca margaretta
PQ374190 Cap Tafarit, Mauritania - Monotheca margaretta
MH212997 Plumularia sp.
MH212994 Plumularia sp.
MH212995 Plumularia sp.
MH212996 Plumularia sp.
DQ855927 Plumularia elongata
MT655150 Plumularia contraria
MT655149 Plumularia contraria
MT655151 Plumularia contraria
MH361355 Plumularia floridana
KX355433 Plumularia habereri
MT655145 Plumularia habereri
MK073072 Plumularia badia
AY787920 Plumularia spiralis
MH108220 Plumularia spiralis
MH108218 Plumularia spiralis
MH108219 Plumularia spiralis
MH108208 Plumularia spiralis
MH108209 Plumularia spiralis
MH108207 Plumularia spiralis
MH108206 Plumularia spiralis
MH108210 Plumularia spiralis
MH108211 Plumularia spiralis
MH108212 Plumularia spiralis
MH108217 Plumularia spiralis
MH108213 Plumularia spiralis
MH108216 Plumularia spiralis
MH108214 Plumularia spiralis
MH108215 Plumularia spiralis
KX355438 Plumularia spiralis
MF000524 Plumularia spiralis
MH212998 Plumularia spiralis
MH108222 Plumularia spiralis
MH108221 Plumularia spiralis
KF982232 Plumularia filicaulis
DQ855926 Plumularia filicaulis
AY787913 Plumularia hyalina
DQ855931 Plumularia setaceoides
KF982240 Plumularia setaceoides
MZ099711 Plumularia cylindrica
KF982224 Plumularia mooreana
KF982228 Plumularia mooreana
KF982226 Plumularia mooreana
PP728274 João Vieira, Guinea Bissau - Plumularia aff. floridana
MH212942 Caribbean, Panama - Plumularia floridana
MH212941 Caribbean, Panama - Plumularia floridana
MH212944 Caribbean, Panama - Plumularia floridana
MH212944 Pacific Ocean, Panama - Plumularia floridana
R MH212939 Pacific Ocean, Panama - Plumularia floridana
MH212947 Pacific Ocean, Costa Rica - Plumularia micronema
MH213000 Pacific Ocean, Panama - Plumularia sp. (cf. propinqua / micronema)
MH212943 Pacific Ocean, Panama - Plumularia floridana
MH212948 Pacific Ocean, Panama - Plumularia micronema
MH212971 Florida, U.S.A. - Plumularia setacea
KF982236 Maldives - Plumularia strictocarpa
KF982233 off Madagascar - Plumularia strictocarpa
MH212957 Pacific Ocean, Panama - Plumularia setacea
MH212967 Pacific Ocean, Panama - Plumularia setacea
KX355441 Martinique - Plumularia strictocarpa
MH212956 Belize - Plumularia setacea
KF982225 French Polynesia - Plumularia strictocarpa
KF982227 French Polynesia - Plumularia strictocarpa
KF982246 La Reunion Island - Plumularia warreni
KF982247 La Reunion Island - Plumularia warreni
KF982245 La Reunion Island - Plumularia warreni
MH212953 Caribbean, Panama - Plumularia setacea
MH212954 Caribbean, Panama - Plumularia setacea
KF982222 Madeira - Plumularia setacea
KF982221 Madeira - Plumularia setacea
KF982220 Madeira - Plumularia setacea
DQ855940 Brazil - Plumularia strictocarpa
MH212938 Caribbean, Costa Rica - Plumularia (cf.) setacea
MH212937 Caribbean, Panama - Plumularia (cf.) setacea
Unpublished Tenerife - Plumularia setacea
MH212972 Florida, U.S.A. - Plumularia setacea
MH212955 Belize - Plumularia setacea
MH212968 Belize - Plumularia setacea
KF982243 La Martinique Island - Plumularia setacea
KF982242 La Martinique Island - Plumularia setacea
MH212978 São Tomé and Principe - Plumularia setacea
MH212990 São Tomé and Principe - Plumularia setacea
MH212979 São Tomé and Principe - Plumularia setacea
MH212980 São Tomé and Principe - Plumularia setacea
PP728275 Unhocomo, Guinea Bissau - Plumularia aff. setacea
MH212989 Azores - Plumularia setacea
MH212966 Azores - Plumularia setacea
JN714622 Azores - Plumularia setacea
MH212991 Azores - Plumularia setacea
JN714621 Azores - Plumularia setacea
MH212965 Azores - Plumularia setacea
MH212950 Berlengas, W Portugal - Plumularia setacea
JN714624 Carvoeiro, S Portugal - Plumularia setacea
MH212982 Azores - Plumularia setacea
KF982218 Italy - Plumularia setacea
JN714623 Naval Club, Azores - Plumularia setacea
KF982202 Mallorca - Plumularia setacea
KF982206 Mediterranean Sea, France - Plumularia setacea
KF982219 Mediterranean Sea, Italy - Plumularia setacea
MG811618 Mediterranean Sea, France - Plumularia setacea
MH212964 Azores - Plumularia setacea
KF982209 South Africa - Plumularia setacea
KF982210 South Africa - Plumularia setacea
KF982211South Africa - Plumularia setacea
EF472684 Chile - Plumularia setacea
EF472682 Chile - Plumularia setacea
EF472683 Chile - Plumularia setacea
EF472685 Chile - Plumularia setacea
MH212934 California, USA - Plumularia cf. duseni
MH212936 California, USA - Plumularia cf. duseni
MH212935 California, USA - Plumularia cf. duseni
KF982238 San Juan Islands, Pacific Ocean, USA - Plumularia setacea
MH212999 California, USA - Plumularia virginiae
KF982229 Argentina - Plumularia duseni
KF982231 Argentina - Plumularia duseni
KT266637 Argentina - Plumularia setacea
KF982230 Argentina - Plumularia duseni
MH212975 California, USA - Plumularia setacea
MH212946 California, USA - Plumularia lagenifera
MH212945 California, USA - Plumularia lagenifera
DQ855928 Washington, USA - Plumularia lagenifera
KF982237 Washington, USA - Plumularia lagenifera
KF982239 Washington, USA - Plumularia lagenifera
KF982249 Washington, USA - Plumularia lagenifera
KP776797 Washington, USA - Plumularia lagenifera
KP776798 Washington, USA - Plumularia lagenifera
MH212973 California, USA - Plumularia setacea
MH212976 California, USA - Plumularia setacea
MH212974 California, USA - Plumularia setacea
KF982204 New Zealand - Plumularia setacea
KF982203 New Zealand - Plumularia setacea
KF982207 New Zealand - Plumularia setacea
AY787887 New Zealand - Plumularia setacea
KF982208 New Zealand - Plumularia setacea
KF982205 New Zealand - Plumularia setacea
FJ550491 South Africa - Plumularia cf. lagenifera
KF982248 La Reunion - Plumularia setacea
KF982244 Europa Island - Plumularia setacea
KF982234 Europa Island - Plumularia setacea
KF982235 Europa Island - Plumularia setacea
KF982215 NW France - Plumularia setacea
JN714620 V. Castelo, N Portugal - Plumularia setacea
KF982216 Plymouth, UK - Plumularia setacea
MH212977 Azores - Plumularia setacea
AY787885 NW France - Plumularia setacea
JN714619 Rinchão, W Portugal - Plumularia setacea
KF982213 NW Spain - Plumularia setacea
KX355447 Norway - Plumularia setacea
FJ550459 NW France - Plumularia setacea
JN714618 Cascais, W Portugal - Plumularia setacea
KF982241 NW France - Plumularia setacea
KF982217 NW France - Plumularia setacea
AY787886 NW France - Plumularia setacea
Unpublished W Portugal - Plumularia setacea
Unpublished W Portugal - Plumularia setacea
Unpublished W Portugal - Plumularia setacea
JN714609 Porto Covo, SW Portugal - Plumularia setacea
JN714610 Sagres, S Portugal - Plumularia setacea
JN714611 Estelas, W Portugal - Plumularia setacea
JN714612 S. Torpes, SW Portugal - Plumularia setacea
JN714613 Ilheu do Soldado, W Portugal - Plumularia setacea
JN714614 Ericeira, W Portugal - Plumularia setacea
JN714615 Sagres, S Portugal - Plumularia setacea
JN714616 Sines, SW Portugal - Plumularia setacea
JN714617 Farilhões, W Portugal - Plumularia setacea
KF982212 NW France - Plumularia setacea
MH212949 Aljezur, SW Portugal - Plumularia setacea
MH212952 Berlengas, W Portugal - Plumularia setacea
Unpublished W Portugal - Plumularia setacea
MH212987 Selvagem Pequena, Portugal - Plumularia setacea
MH212986 Azores - Plumularia setacea
MH212961 Desertas, Portugal - Plumularia setacea
MH212958 Azores - Plumularia setacea
MH212960 Azores - Plumularia setacea
MH212959 Azores - Plumularia setacea
JN714608 Gorringe - Plumularia setacea
MH212992 Azores - Plumularia setacea
MH212988 Selvagem Pequena, Portugal - Plumularia setacea
MH212983 Selvagem Grande, Portugal - Plumularia setacea
MH212985 Azores - Plumularia setacea
MH212984 Selvagem Grande, Portugal - Plumularia setacea
JN714607 Farilhões, W Portugal - Plumularia setacea
JN714606 Azores - Plumularia setacea
JN714605 Azores - Plumularia setacea
KF982223 Madeira - Plumularia setacea
MH212962 Azores - Plumularia setacea
MH212969 Azores - Plumularia setacea
MH212963 Azores - Plumularia setacea
KF982214 NW France - Plumularia setacea
KF982201 W France - Plumularia setacea
MH212970 Algarve, S Portugal - Plumularia setacea
MH212951 Berlengas, W Portugal - Plumularia setacea
MH212981 Sagres, S Portugal - Plumularia setacea
MH212993 Azores - Plumularia setacea
Unpublished W Portugal - Plumularia setacea
Tree scale: 0.1
